# Isolation and Molecular Characterization of a Novel Lytic Bacteriophage That Inactivates MDR *Klebsiella pneumoniae* Strains

**DOI:** 10.3390/pharmaceutics14071421

**Published:** 2022-07-06

**Authors:** Victor M. Balcão, Fernanda C. Moreli, Erica C. Silva, Bianca G. Belline, Layla F. Martins, Fernando P. N. Rossi, Carla Pereira, Marta M. D. C. Vila, Aline M. da Silva

**Affiliations:** 1 PhageLab, Laboratory of Biofilms and Bacteriophages, University of Sorocaba, Sorocaba 18023-000, Brazil; femoreli@yahoo.com.br (F.C.M.); erica.silva@edu.uniso.br (E.C.S.); biancagbelline@gmail.com (B.G.B.); marta.vila@prof.uniso.br (M.M.D.C.V.); 2 Department of Biology and CESAM, Campus Universitário de Santiago, University of Aveiro, 3810-193 Aveiro, Portugal; csgp@ua.pt; 3 Department of Biochemistry, Institute of Chemistry, University of São Paulo, São Paulo 05508-000, Brazil; layla@iq.usp.br (L.F.M.); fernandorossi@usp.br (F.P.N.R.); almsilva@iq.usp.br (A.M.d.S.)

**Keywords:** bacteriophage, *Demerecviridae*, *Sugarlandvirus*, *Klebsiella pneumoniae*, antibacterial phage activity

## Abstract

The worldwide increase in serious infections caused by multidrug-resistant (MDR) *K. pneumoniae* emphasizes the urgent need of new therapeutic strategies for the control of this pathogen. There is growing interest in the use of bacteriophages (or phages) to treat *K. pneumoniae* infections, and newly isolated phages are needed. Here, we report the isolation and physical/biological/molecular characterization of a novel lytic phage and its efficacy in the control of MDR *K. pneumoniae*. The phage vB_KpnS_Uniso31, referred to hereafter as phage Kpn31, was isolated from hospital wastewater using *K. pneumoniae* CCCD-K001 as the host. Phage Kpn31 presents a siphovirus-like morphotype and was classified as *Demerecviridae*; *Sugarlandvirus* based on its complete genome sequence. The 113,444 bp Kpn31 genome does not encode known toxins or antimicrobial resistance genes, nor does it encode depolymerases related sequences. Phage Kpn31 showed an eclipse time of 15 min and a burst size of 9.12 PFU/host cell, allowing us to conclude it replicates well in *K. pneumoniae* CCCD-K001 with a latency period of 30 min. Phage Kpn31 was shown to be effective against at least six MDR *K. pneumoniae* clinical isolates in in vitro antibacterial activity assays. Based on its features, phage Kpn31 has potential for controlling infections caused by MDR *K. pneumoniae.*

## 1. Introduction

The worldwide emergence of infections caused by multidrug-resistant (MDR) pathogens is a terrifying problem for human health and is predicted to cause 10 million antimicrobial resistance-related deaths per year globally by 2050 [1]. The most common multidrug-resistant pathogens, *Enterococcus faecium*, *Staphylococcus aureus*, *Klebsiella pneumoniae*, *Acinetobacter baumannii*, *Pseudomonas aeruginosa,* and *Enterobacter* spp., which have been integrated within the acronym “ESKAPE”, are the leading cause of hospital-acquired infections throughout the world [2,3]. For instance, *K. pneumoniae* accounts for approximately 3–8% of all nosocomial bacterial infections in the USA [4]. *K. pneumoniae* causes infections in the urinary tract, respiratory tract, lung, wound sites, and blood, mainly in individuals with debilitating diseases, typically colonizing the surface of the nasopharynx and gastrointestinal tract mucosa [5]. Various virulence factors contribute to the infectivity of *K. pneumoniae* strains, including their capsular polysaccharides, lipopolysaccharides, and exopolysaccharides, which provides a protection against the host immune response [6,7]. Moreover, *K. pneumoniae* forms a biofilm and displays intrinsically and acquired resistance to several antibiotics [8].

The increasingly high percentage of antimicrobial-resistant *K. pneumoniae* reflects the continuous loss of effectiveness of the antimicrobial therapies and emphasizes the need for the prudent use of antibiotics, as treatment options for MDR bacterial infections are diminishing [8,9,10], and the discovery of new antibiotics for MDR Gram-negative ESKAPE pathogens remains largely elusive [11]. Hence, new therapeutic and prophylactic strategies for the control of MDR *K. pneumoniae* infection are needed, such as the combined use of antibiotic therapies, antibacterial antibodies, enzyme-based antibacterials, and phage therapy [12,13]. Indeed, human patients and animal models with *K. pneumoniae* infections have been successfully treated with phage therapy, combined or not with conventional antibiotic strategies [14,15,16,17,18,19,20,21,22,23].

In contrast with conventional antibacterial therapies, phage therapy seems not to damage the host commensal microbiota, presents a lower toxicity, and has relatively low cost, among other advantages [24]. Newly isolated phages are needed to meet the growing interest in the use of phages to treat *K. pneumoniae* infections [23]. Therefore, the aim of the study reported herein was to isolate (from hospital wastewater) and select a phage based on its ability to form clear lysis plaques, to characterize (regarding physical, biological, and genome sequence characteristics) and evaluate its in vitro efficiency to control MDR *K. pneumoniae* clinical isolates, in order to assess its potential application in the treatment of infections caused by MDR K. *pneumoniae*.

## 2. Materials and Methods

### 2.1. Bacterial Strains and Culture Conditions

Lyophilized stocks of *K. pneumoniae* CCCD-K001, *Escherichia coli* CCCD—E003, *Salmonella enterica* CCCD—S004, *Pseudomonas aeruginosa* CCCD—P004, *Proteus mirabilis* CCCD—P001, *Enterococcus faecalis* CCCD—E002, *Bacillus subtilis* CCCD—B010, *Staphylococcus epidermidis* CCCD—S010, *Staphylococcus aureus* CCCD—S009, and *Acinetobacter baumanii* ATCC 16606 were purchased from Cefar Diagnóstica (São Paulo, SP, Brazil).

Bacterial suspensions (Brucella Broth containing 15% (*v/v*) glycerol) of clinical isolates of *K. pneumoniae* were provided by Dr. Priscilla Carmona, Technical Coordinator of the Laboratory of Microbiology and Urinalysis of Hospital UNIMED Miguel Soeiro, Sorocaba, SP, Brazil.

Bacteria were cultured on Tryptic Soy Agar (TSA, Sigma-Aldrich, St. Louis, MO, USA) and incubated for 24 h at 37 °C. One isolated colony was aseptically picked from the TSA plate, resuspended in 5 mL of sterile Tryptic Soy Broth (TSB, Gibco Diagnostics, Madison, WI, USA), and incubated at 37 °C under orbital shaking at 100 rpm until reaching log phase or stationary phase (up to 24 h).

### 2.2. Antibiotic Susceptibility Assays

Overnight cultures (100 µL) of *K. pneumoniae* strains in TSB were mixed with 4 mL of molten top agar TSB (MTA-TSB) in a 15 mL test tube, and were tapped gently and poured onto an agar plate with TSA, which was then gently swirled. The plates were allowed to dry for 1–2 min, after which the antibiotic discs were gently placed using sterile tweezers. The discs impregnated with antibiotics at the indicated concentration (nalidixic acid 30 µg, amikacin 30 µg, amoxicillin/clavulanic acid 20/10 µg, ampicillin 10 µg, cephalothin 30 µg, cefepime 30 µg, ceftriaxone 30 µg, cefuroxime 30 µg, cefuroxime acetyl 30 µg, ciprofloxacin 5 µg, ertapenem 10 µg, gentamicin 10 µg, meropenem 10 µg, nitrofurantoin 300 µg, norfloxacin 10 µg, piperacillin/tazobactam 100/10 µg, trimethoprim/sulfamethoxazole 25 µg, ceftazidime 30 µg, colistin 10 µg, imipenem 10 µg, tigecycline 30 µg, ampicillin/sulbactam 10/10 µg, and cefoxitin 30 µg) were purchased from Diagnósticos Microbiológicos Especializados (Araçatuba, SP, Brazil).

### 2.3. Phage Enrichment, Isolation and Enumeration

Phage enrichment from hospital wastewater was performed according to Harada et al. [25]. A wastewater/sewage sample (50 mL) collected from Hospital UNIMED Miguel Soeiro, Sorocaba, SP, Brazil on 16 January 2018 and 22 February 2018, was mixed with an overnight culture of *K. pneumoniae* CCCD-K001 (50 μL) and supplemented with 50 mL of TSB. After incubation at 37 °C for 24 to 48 h, the enriched sample was centrifuged (9000× *g*, 4 °C, 10 min), and the supernatant was collected and filtered through a sterile 0.22 μm filtration system (Stericup™-GP, Merck-Millipore, Darmstadt, Germany).

The presence of phages in the enriched supernatant was verified by spot-testing. Ten microliter droplets of filtered supernatant were poured onto a lawn of the *K. pneumoniae* CCCD-K001, the plates were left to dry out and were subsequently incubated at 37 °C during the night. After the incubation period, the plates were checked for either clear or turbid lysis zones, indicative of the presence of phages.

Isolation of phage plaques was carried out using the conventional double-layer agar method according to Harada et al. [25], Silva et al. [26], and Adams [27]. A *K. pneumoniae* CCCD-K001 overnight culture (100 µL) was mixed with 5 mL of molten top agar TSB (MTA-TSB) in test tubes and a phage suspension prepared from a single plaque resuspended in TSB, tapped gently, and poured onto TSA plates which were gently swirled and allowed to dry out for 1–2 min, followed by overnight incubation at 37 °C. The procedure was repeated until all phage plaques exhibited the same morphology. The plates were stored at 4 °C as phage stocks.

Titres of phage suspensions (PFU/mL (plaque forming units/mL)) were determined by plating serially diluted phage suspensions using the double-layer agar method described above.

### 2.4. PEG Precipitation of Phage Suspensions

High titre (10^13^ PFU/mL) phage suspensions were concentrated by mixing (2:1, *v/v*) with 10% (*w/w*) polyethylene glycol 8000 (PEG 8000, Sigma-Aldrich, St. Louis, MO, USA) and 1 M NaCl (Labsynth, Diadema, SP, Brazil), and were incubated overnight at 4 °C. The mixture was then centrifuged at 11,000 rpm at 4 °C for 45 min, the supernatant was discarded, and the pellet was resuspended in 5 mM MgSO_4_ (Labsynth, Diadema, SP, Brazil).

### 2.5. Transmission Electron Microscopy (TEM)

PEG-concentrated phage suspensions were negatively stained with uranyl acetate (Sigma-Aldrich, St. Louis, MO, USA). Glow-discharged carbon film-coated 3-mm copper grids (Ultrathin Formvar/Carbon on a 400-mesh copper grid, CF400-Cu, Electron Microscopy Sciences, Hatfield, PA, USA) were sequentially dipped in droplets of (i) concentrated phage suspension (15 min), (ii) ultrapure water (3× droplets, 10 s in each), (iii) uranyl acetate (2%, *w/v*; pH 7.0) (5 min). The grids were then imaged out in a transmission electron microscope from JEOL (model JEM 1011, Tokyo, Japan), at an acceleration voltage of 60 kV using a CCD camera from GATAN Inc. (model 830 J46W44, serial no. 11042801W0830, Pleasanton, CA, USA).

### 2.6. Purification of Phage DNA and Whole Genome Sequencing

The PEG-concentrated phage suspension (500 µL) was treated with 1.25 µL DNAse-I (20 mg/mL, Transgen Biotech, Beijing, China) and 1.25 µL RNAse (10 mg/mL, Transgen Biotech, Beijing, China) at 37 °C for 1 h. Following incubation, 1.25 µL Proteinase K (Transgen Biotech, 20 mg/mL), 25 µL aqueous SDS (Sigma-Aldrich, St. Louis, MO, USA) (10%, *w/w*) (final SDS concentration of 0.5%, *w/w*), and 20 µL aqueous EDTA (Sigma-Aldrich, St. Louis, MO, USA) (0.5 M, pH 8.0) (final EDTA concentration of 20 mM) were added to the samples, followed by incubation at 60 °C for 1 h, after which the mixture was allowed to cool down to room temperature. DNA extraction was performed using phenol/chloroform (Sigma-Aldrich, St. Louis, MO, USA) protocol, as briefly described. Phenol was added in a 1:1 (*v/v*) proportion. After centrifugation at 6000 rpm for 5 min, an equal volume of chloroform was added to the supernatant, and this last step repeated twice. After centrifugation (6000 rpm for 5 min), the aqueous phase was collected. DNA was precipitated by mixing the supernatant to 1/10 volume 3 M NaOAc (Sigma-Aldrich, St. Louis, MO, USA) (pH 7.5) and 2.5× volume of cold absolute ethanol (Sigma-Aldrich, St. Louis, MO, USA). The mixture was incubated overnight at −20 °C, at −86 °C for 30 min, and was centrifuged at 14,000 rpm for 20 min. The pellet was allowed to dry, after which it was dissolved in 50 µL nuclease-free ultrapure water (ThermoScientific, Waltham, MA, USA). Purified phage DNA was subjected to a final clean-up step using QIAamp mini spin columns (Qiagen, Germantown, MD, USA) and was stored at −20 °C. DNA purity and concentration were evaluated on a ND-1000 spectrophotometer (NanoDrop Technologies Inc., Wilmington, DE, USA) at 260 nm, 280 nm, and 230 nm. Further quantification was performed with Quant-iT Picogreen dsDNA assay kit (Life Technologies, Carlsbad, CA, USA). The DNA integrity was examined with a DNA 7500 chip using a 2100 Bioanalyzer (Agilent, Palo Alto, CA, USA).

Purified phage DNA (20–30 ng) was used to prepare the shotgun genomic library with an Illumina Nextera DNA library preparation kit (Illumina, San Diego, CA, USA). The DNA fragment library was cleaned up with Agencourt AMPure XP beads (Beckman Coulter, Indianapolis, IN, USA) and the average fragment size (400–700 bp) was verified by running in the 2100 Bioanalyzer using an Agilent High Sensitivity DNA chip (Agilent, Palo Alto, CA, USA). Quantification of the Illumina sequencing library, normalization, and sequencing were performed following standard protocols for sequencing in the Illumina MiSeq platform. The library was subjected to one run using the MiSeq Reagent kit v3 (600-cycle format, paired-end (PE) reads).

### 2.7. Phage Genome Assembly and Annotation

Raw PE sequencing reads data were quality inspected and as the quality of the R1 reads was higher than that of the R2 reads, only R1 reads were used for genome assembly. R1 reads were trimmed and quality filtered using fastp [28], with a sliding window quality cutoff of Q15. Bacterial host residual contaminant sequences were filtered out by mapping R1 reads against a *K. pneumoniae* complete genome (Genbank accession number NC_016845.1) using BBSLPIT tool (version 38.18). The filtered reads were de novo assembled with SPADES using different kmer lengths (with the flag -k 21, 33, 55, 77, 99, 113, 121, and 127) (version 3.13.1) [29]. BOWTIE2 (version 2.3.4.1) [30] was used to map the reads to the genome assemblies. The assembled phage genome was inspected for repeat sequences using the Generic Repeat Finder [31].

The phage genome sequence was annotated with the Hmmer tool (version 3.3.2) using the pVOGs [32] and PHROG [33] databases (downloaded in their last version on 15 April 2022, in the HMM aminoacid profile). The tool DeepCapTail [34] was used to predict viral structural proteins (capsid and tail). A circular map of the annotated phage genome was generated using CG view (version 2.0.2) [35].

### 2.8. Proteome-Based Clustering and Phylogenetic Analysis

A subset of 233 phage genomes close to the phage Kpn31 genome was selected from the complete Millard Lab phage genome database (~21,000 genomes) [36], whoch was downloaded in January 2022. The selection was based on the frequency of kmers with a distance of ≤0.5 using the Mash tool [37]. This subset of 233 genomes was subjected to shared proteome clustering analysis using the vConTACT2 tool [38]. The resulting network graph was visualized and annotated with Cytoscape (v3.8.0) [39].

A core of protein clusters was retrieved from the phages belonging to the Kpn31 cluster, each protein sequence from each phage was aligned, concatenated, and the result was used to calculate a maximum likelihood phylogenetic tree using the FastTree [40] with 1000 bootstraps. The resulting tree was visualized using the FigTree (https://github.com/rambaut/figtree/releases/tag/v1.4.4 (accessed on 26 May 2022)) program.

### 2.9. Evaluation of the Host Range

The phage host range was evaluated with 39 bacterial strains using spot testing. Briefly, 4 mL of MTA-TSB were added with 300 μL of bacterial culture and overlaid on solid TSA, which were allowed to dry out and were spotted with 10 μL-droplets of serial diluted phage suspensions, and were then incubated at 37 °C for 24 h. Bacterial sensitivity to the phage was inferred from the presence of clear lysis (+) or no-lysis (−) zones where the phage suspension was spotted. In bacteria in which the occurrence of lysis zones was observed, the plating efficiency was determined.

The efficiency of plating (EOP) was calculated based on the relative number of plaques that a phage stock suspension produces on a certain bacterial strain, using the double agar-layer method described above. After incubation at 37 °C for 24 h, the number of PFUs/strain was determined. The EOP for each bacterial strain was calculated considering an EOP value of 100% for *K. pneumoniae* CCCD-K001 (strain used for phage isolation). The values displayed for the EOP are the mean of three independent determinations. The EOP was scored as high, moderate, or low, when the relative phage titres for each strain represented 50% or more, 10–0.1%, and 0.1–0.001%, respectively, of the PFUs found for the primary host. An EOP equal to or under 0.001 was classified as inefficient [41].

### 2.10. One-Step Growth Curve (OSGC)

The growth parameters for phage Kpn31 were obtained from the OSGC using *K. pneumoniae* CCCD-K001 and Kpn31 at MOI ≤ 0.001, as described by Harada et al. [25], with three independent experiments. The experimental results were then plotted to determine the eclipse, latent, and intracellular accumulation periods and the burst size of the phage. A typical sigmoidal (or four-parameter logistic regression, 4-PL) model ($\mathrm{Log}\;\left(P_{t}\right)=\;\beta +\frac{\alpha -\beta }{1+{\left(\frac{t}{\eta }\right)}^{\gamma }}$) was then adjusted to the OSGC experimental data via nonlinear fitting, where P_t_ represents the concentration of phage (PFU/mL) at time t, α is the phage concentration at t = 0, β is the phage concentration at t = ∞, η is the inflection point of the sigmoidal curve, γ is the Hill’s slope (steepness) of the curve, and t is the time (min). Nonlinear fitting of the phage growth data to the 4-PL model was carried out using the Solver function of Microsoft Excel (Microsoft, Redmond, WA, USA).

### 2.11. Adsorption Rate

The phage adsorption rate was determined as described by Harada et al. [25], with three independent assays. Phage adsorption was expressed as the decrease of phage titre in the supernatant (%) compared with the time zero. Assuming that the phage particles have the ability to adsorb onto susceptible bacterial cells and form a reversible phage-bacteria complex that may or may not lead to an infected bacterium, then the model $\mathrm{Free}\;\mathrm{phage}\;\left(P\right)+\mathrm{bacteria}\;\left(X_{0}\right)\;\begin{array}{c} \delta \cdot X_{0}\\ ⇄\\ \phi \end{array}\;\mathrm{Reversible}\;\left\{\mathrm{phage}-\mathrm{bacteria}\right\}\;\mathrm{complex}\;\left(\Delta \right)$, postulated by Watanabe et al. [42] and Moldovan et al. [43], leads to the set of equations $\{\begin{array}{c} \frac{\mathrm{dP}}{\mathrm{dt}}=-\delta \cdot P\cdot X_{0}+\phi \cdot \Delta \\ P+\Delta =1.0 \end{array}$, whose solution yields the mathematical model $\frac{P_{t}}{P_{0}}=\frac{\phi }{\delta \cdot X_{0}+\phi }\left\{1+\frac{\delta \cdot X_{0}}{\phi }\cdot e^{-\phi \left(\frac{\delta \cdot X_{0}+\phi }{\phi }\right)\cdot t}\right\}$ [42,44], which was used to estimate the adsorption rate via nonlinear fitting to the experimental data, where P_t_ and P_0_ represent the phage concentrations (PFU mL^−1^) at times t and 0, respectively, δ represents the (first order) phage adsorption rate onto susceptible bacterial host cells (PFU^−1^ CFU^−1^ mL^−1^ min^−1^), ϕ represents the (first order) phage desorption rate from reversible phage-bacteria complexes (mL min^−1^), X_0_ represents the initial concentration of uninfected (susceptible) bacterial cells (CFU/mL), and t is the infection time (h). Nonlinear fitting of the phage adsorption data to the negative exponential model was carried out using the Solver function of Microsoft Excel (Microsoft, Redmond WA, USA).

### 2.12. Bacterial Kill Assay

Inactivation of planktonic bacterial cells (10^5^ CFU/mL) by phage Kpn 31 was studied at MOI 1 (10^5^ PFU/mL) and MOI 1000 (10^8^ PFU/mL). Control assays were performed with no phage added (bacterial control (BC)) or no bacterium added (phage control (PC)). Controls and test samples (bacterium plus phage (BP)) were incubated under the same conditions, and aliquots were withdrawn after 0, 2, 4, 6, 8, 10, 12 and 24 h of incubation. In all of the assays, the phage titre was determined in triplicate using the double agar-layer method, followed by incubation for 24 h at 37 °C. The bacterial concentration was determined in triplicate in a solid TSA medium by plating 10 µL, followed by an incubation period at 37 °C for 24 h. Three independent assays were performed.

### 2.13. UV-Vis Spectral Scans

The spectrophotometric assay was performed according to the procedure described elsewhere [25,45]. Five dilutions of PEG-concentrated phage suspensions were prepared in an SM phage buffer (200 mM NaCl_2,_ 10 mM MgSO_4_, 50 mM Tris-HCl, pH 7.5) up to a final volume of 2000 µL, and absorbance was determined at 254.5 nm and 320 nm.

### 2.14. X-ray Diffraction Analysis

X-ray diffractograms of PEG-concentrated phage suspensions were obtained in an X-ray Diffractometer (XRD) from Shimadzu (model XRD7000, Kyoto, Japan), using X-ray radiation filtered through a Cu target. X-ray scanning was carried out at diffraction angles of 2-Theta (5–90°, increments of 0.02 degrees and rate of 2° min^−1^), at a voltage of 40 kV, electric current of 30 mA, and X-ray power of 3 kW. The data from X-ray diffractograms were used in the Scherrer equation [46] ($\tau \left(\mathrm{nm}\right)=\frac{Κ\times \lambda }{FWHM\times \cos\left(\theta \right)}$), where *τ* and *λ* have nm as the units of measurement, *FWHM* is the full width at the half maximum of the peak (rad), Κ is a dimensionless shape factor (0.94), and θ is half of Bragg angle (rad). The Scherrer equation is useful for qualitative comparisons, and only for crystallites smaller than 1000 Å.

### 2.15. Statistical Analyses

Statistical analysis of the experimental data was carried out using GraphPad Prism 7.04 (GraphPad Software, San Diego, CA, USA). The experimental data were assessed for normal distribution using the Kolmogorov–Smirnov test, whereas the homogeneity of the variance was assessed using the Levene’s test. The significance of bacterial and phage concentrations between MOI values and along the assays was determined using a two-way analysis of variance (ANOVA) and the Bonferroni post hoc test. For different MOI values, the significance of the differences was evaluated by comparing the results produced in the test samples with the results produced for the corresponding control samples, for the different inactivation times. *p*-values < 0.05 were considered statistically significant.

## 3. Results

### 3.1. Phage Isolation and Purification

**Figure 1:**
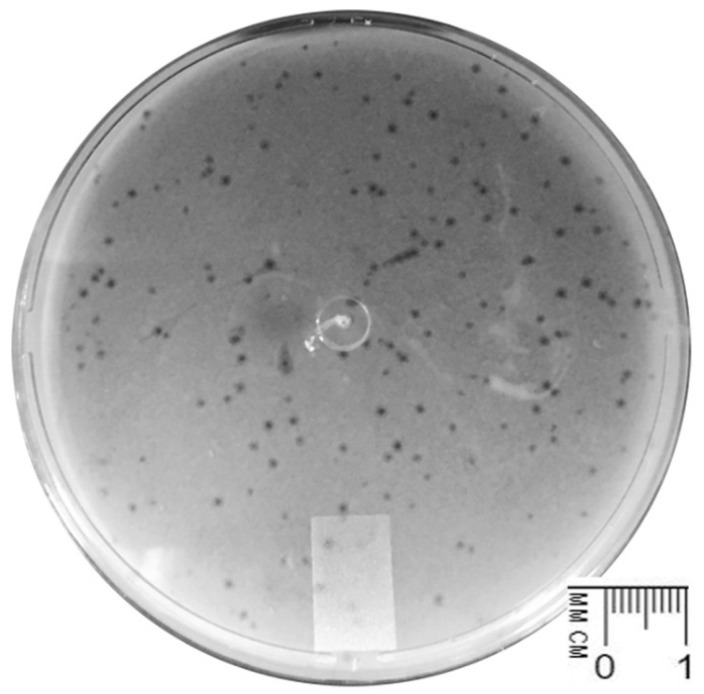
Lysis plaques of phage vB_KpnS_Uniso31 (Kpn31) in a *K. pneumoniae* CCCD-K001 lawn. Plaques present the same morphology without secondary halos around clear zones of lysis.

One phage clear lysis plaque was selected among the clear and turbid plaques of the same size obtained from the enrichment of a wastewater sample (sewage) collected at Hospital UNIMED Miguel Soeiro, Sorocaba, São Paulo, Brazil, using *K. pneumoniae* CCCD-K001 as the host strain. This is a commercial collection strain derived from human urine clinical isolate, and it is reported as an MDR strain [unpublished]. After three rounds of purification on a *K. pneumoniae* CCCD-K001 lawn, the isolated phage produced clear and translucent plaques with diameters of ≈1.0 mm (Figure 1). The isolated phage was named vB_KpnS_Uniso31, hereafter referred to as Kpn31. High concentration suspensions (10^13^ PFU/mL) of phage Kpn31 were obtained for further assays, aiming at its characterization.

### 3.2. Virion Morphology

**Figure 2:**
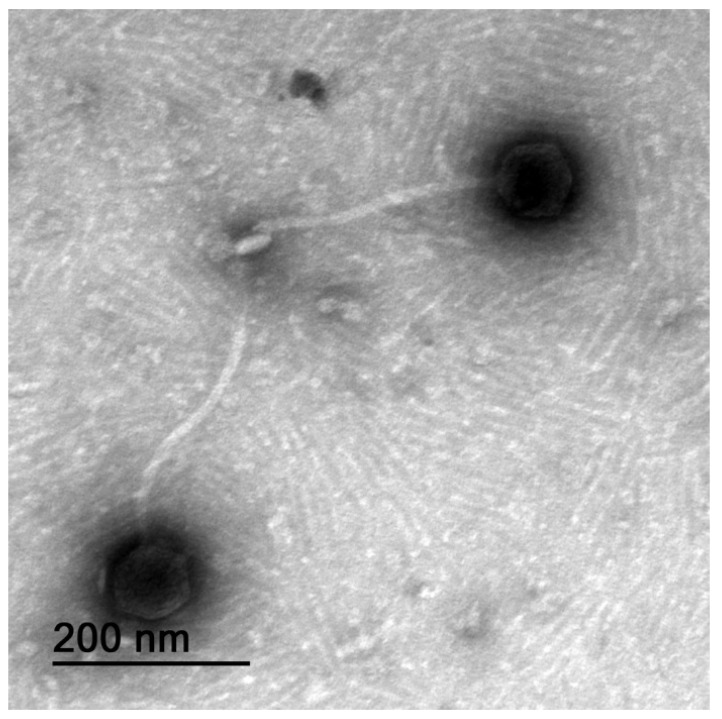
Negative-staining TEM photomicrograph of phage Kpn31 at ×100,000 magnification, allowing to clearly observe the nucleocapsid, the non-contractile thin tail, the tail tube, and the central tail spike at the distal part of the tail.

The TEM photomicrograph (Figure 2) clearly shows that the Kpn31 virion has a polyhedral head and a long flexible, non-contractile tail of the *Siphoviridae*-like tail morphotype [47] belonging to the Caudovirales order of dsDNA viruses.

### 3.3. Genomic Characterization of Phage Kpn31

The genome of phage Kpn31 was sequenced and assembled, resulting in a contig of 113,444 bp. The contig had 141 bp direct terminal repeats, indicating that the assembled phage genome is complete. The GC content of the Kpn 31 genome is 45.3%, whereas that of the *K. pneumoniae* is 57.5% [48]. The overall characteristics of the assembly and of the genome annotation are summarized in Table 1.

**Figure 3:**
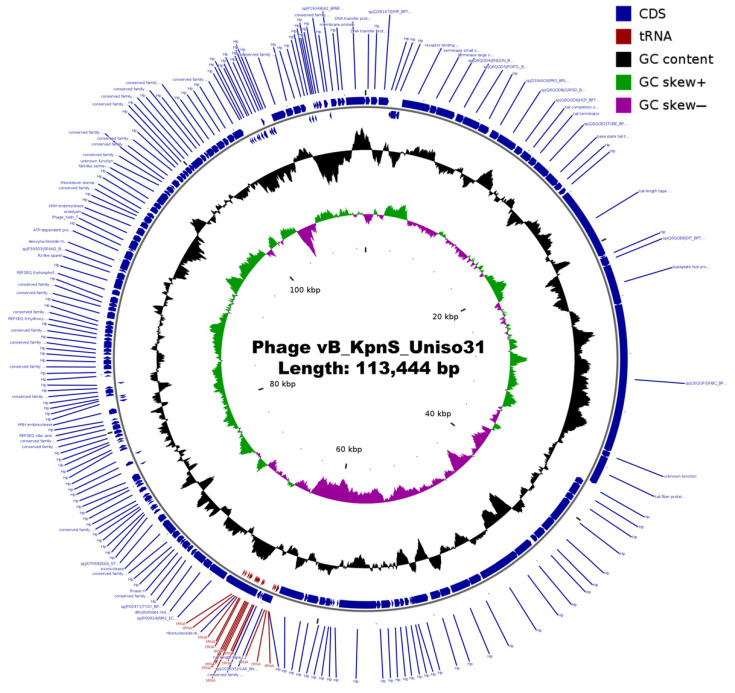
Annotated genome map of phage Kpn31. GC skew, G + C content, and predicted CDS are shown. The middle ring indicated the GC content (black), and the innermost ring represented the GC skew of Kpn31 genome. Blue arrows in the outer ring are annotated coding sequences (CDSs) numbered according to the annotation in Supplementary Table S1. Red arrows correspond to tRNAs. The arrows represent the direction of transcription (strand + or −).

The genome of phage Kpn31 encodes 14 tRNAs and 188 protein coding genes (CDS, coding sequences) (Supplementary Table S1). A comparison of the annotated CDS with different databases revealed that 144 CDS are predicted as proteins of an unknown function or hypothetical proteins. Nevertheless, further investigation suggests that 54 of the hypothetical proteins have a predicted functional description, mostly as capsid or tail proteins (Supplementary Table S1). Several structural proteins, such as capsid, tail, and baseplate proteins, were annotated along with DNA metabolism-related proteins and host lysis proteins (holin, endolysin, and spanin). We did not detect genes related to depolymerases, toxins, virulence factors, antibiotic resistance, or integrases among the Kpn31 CDS with predicted functions (Supplementary Table S1). A circular map of the annotated Kpn31 genome is shown in Figure 3.

**Figure 4:**
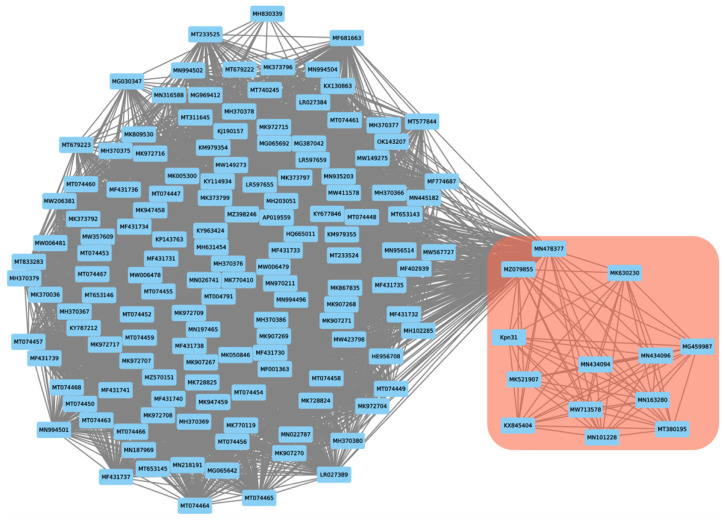
Proteome-based network analysis calculated with vConTACT2. The predicted proteome of phage Kpn31 was clustered with the proteome of its closest 233 annotated phages pre-selected from the Millard phage database. The Kpn31 cluster encompassing the 12 phages listed in Table 2 is highlighted by a red box. The numbers in the nodes correspond to the NCBI accession of each phage genome. The edges between nodes represent shared proteins.

The proteome clustering and network analysis performed with vConTACT2 [38] assigned the Kpn31 genome to a viral cluster with 12 phages (Figure 4 and Table 2), all of them currently classified as *Demerecviridae* or *Sugarlandvirus*. Therefore, phage vB_KpnS_Uniso31 (phage Kpn31) can be assigned to the *Sugarlandvirus* genus. The taxonomic classification of Kpn31 agrees with the TEM analysis (Figure 2), which showed a siphovirus-like morphotype, which is typical of *Demerecviridae* phages such as the T5 coliphage [47]. Moreover, the Kpn31 genome nucleotide sequence is greatly similar (~95%) to most of the 12 phage genomes that clustered together in the proteome network analysis (Table 2).

**Figure 5:**
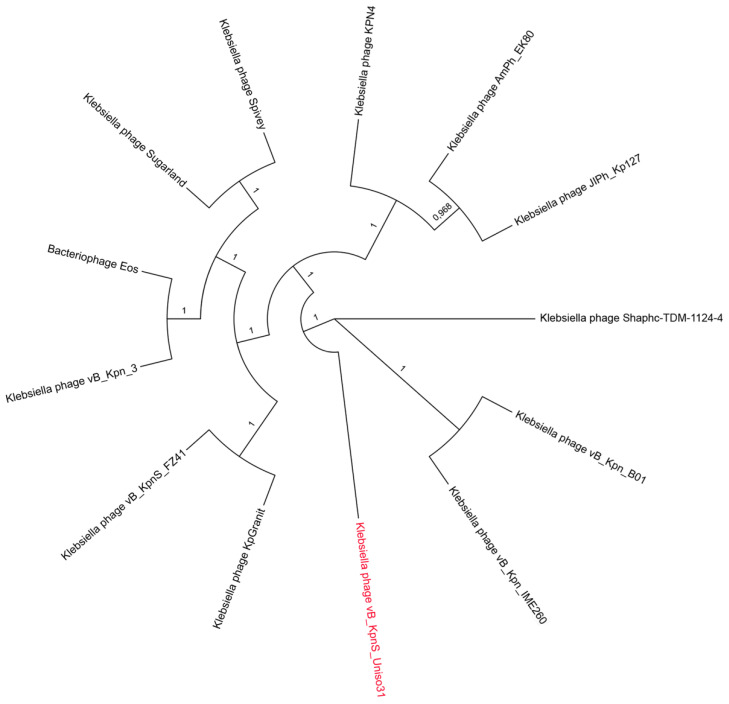
Phylogenetic tree calculated using the core protein clusters from 12 phages connected with phage vB_KpnS_Uniso31 (highlighted in red). Sequences of protein clusters were used for a maximum likelihood (ML) phylogenetic reconstruction using 1000 bootstrap replicates.

The core protein clusters from the 12 phages determined as the closest to Kpn31 by the network analysis (Table 2), along with Kpn31 itself (Supplementary Table S2), were used for the phylogenetic analysis. While some phages are grouped, such as phages Sugarland and Spivey, phages Shaphc-TDM-1124-4 and Kpn31 appear isolated in an branch (Figure 5).

### 3.4. Host Range of Phage Kpn31

The susceptibility of 39 bacterial strains to phage Kpn31 was inferred by spot test (Table 3). As expected, bacterial species such as *E. coli*, *S. enterica*, *P. aeruginosa*, *P. mirabilis*, *E. faecalis*, *B. subtilis*, *S. epidermidis*, *S. aureus,* and *A. baumanii* were not susceptible to phage Kpn31. On the other hand, 11 out of 26 strains of *K. pneumoniae* derived from clinical isolates were susceptible to Kpn31 on spot test (Table 3), forming completely cleared zones. The EOP for these 11 *K. pneumoniae* clinical isolates in relation to *K. pneumoniae* CCCD-K001 varied from 89.21% to 0.9% (Table 3). Strains 1, 2, 4, 5, 13, and 24 presented EOPs >50% and <90%.

It is important to mention that all *K. pneumoniae* clinical isolates evaluated in the host range tests were found to be resistant to many antibiotics exclusively utilized in hospital settings (Table S3).

### 3.5. Determination of Burst Size and Phage Adsorption Rate

**Figure 6:**
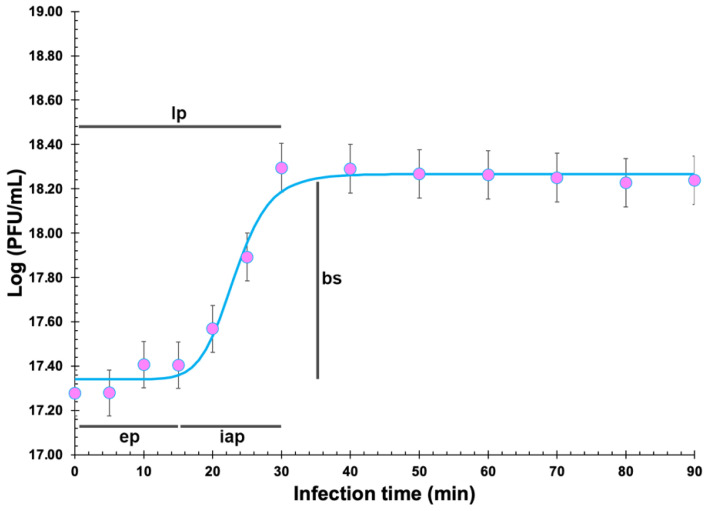
One-step growth curve of phage Kpn31 using *K. pneumoniae* CCCD-K001 as the host. The assay was performed with *K. pneumoniae* CCCD-K001 in the late exponential phase (10^8^ CFU/mL) and phage Kpn31 at a titre of 10^5^ PFU/mL (MOI ≤0.001). The phage growth data were adjusted via nonlinear fitting to a typical sigmoidal (or four-parameter logistic regression) model to estimate the phage eclipse (ep), latent (lp), and intracellular accumulation (iap) periods and burst size (bs) as indicated. Values are the means of three independent assays. Error bars represent the standard deviations. lp: 30 min; ep: 15 min; iap: 15 min; bs: 9.12 PFUs/host cell.

The phage eclipse, latent, and intracellular accumulation periods and burst size were estimated as being 15 min, 30 min, 15 min, and 9.12 PFUs/host cell (Figure 6), respectively.

**Figure 7:**
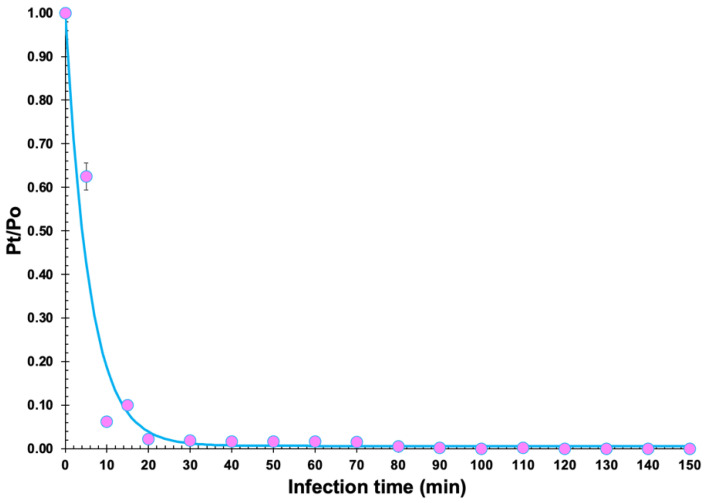
Adsorption curve of the phage Kpn31 onto *K. pneumoniae* CCCD-K001 cells, displaying the non-linear fitting of a negative exponential function to the experimental data. Values represent the means of three independent assays. Error bars represent the standard deviations. The values of the parameters obtained from the nonlinear fitting of the adsorption model to the experimental data were δ = 1.700 × 10^−9^ PFU^−1^ CFU^−1^ mL^−1^ h^−1^ (phage adsorption rate) and *φ* = 0.00110 h^−1^ (phage desorption rate) (X_0_ = 1.0 × 10^8^ CFU/mL).

The ability of phage Kpn31 to bind to *K. pneumoniae* CCCD-K001 cells was evaluated by the adsorption assay displayed in Figure 7. The adsorption rate was estimated via nonlinear fitting of the phage adsorption model to the experimental data, producing a value for the adsorption rate of the phage particles equal to δ = 1.700 × 10^−9^ PFU^−1^ CFU^−1^ mL^−1^ h^−1^ and a value for the phage desorption rate equal to *φ* = 0.00110 h^−1^ (X_0_ = 1.0 × 10^8^ CFU/mL). Thus, within 10 min after phage-host mixture, ~90% of the phage particles were adsorbed to the bacterial cells (Figure 7).

### 3.6. In Vitro Inactivation of K. Pneumoniae CCCD-K001

**Figure 8:**
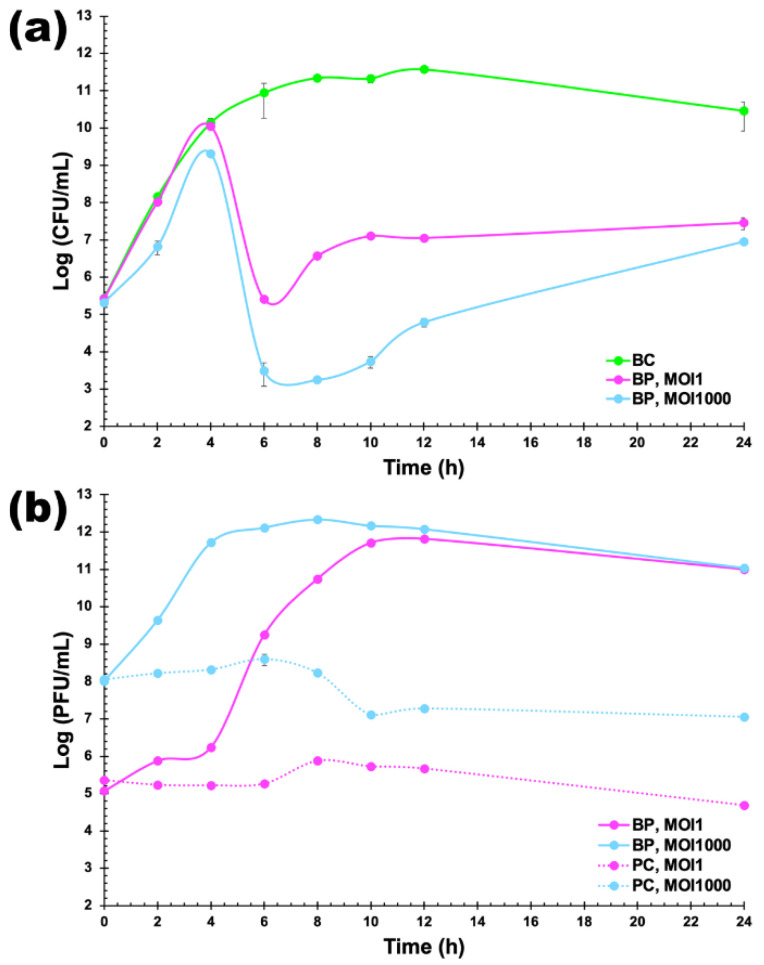
In vitro inactivation of *K. pneumoniae* by phage Kpn31. (**a**) Inactivation of *K. pneumoniae* CCCD-K001 was evaluated at a MOI of 1 and of 1000 by determining the bacterial concentration (CFU/mL) (**a**) and phage titre (PFU/mL) (**b**) during 24 h. BC, bacterial control (no phage added); BP, bacteria with phage; PC, phage control. Values represent the mean of three independent assays, whereas error bars represent the standard deviation.

The in vitro inactivation of *K. pneumoniae* CCCD-K001 by phage Kpn31 was assessed by means of bacterial kill curves at a MOI of 1 and of 1000 (Figure 8a). The maximum inactivation of *K. pneumoniae* with phage Kpn31 was 5.6 and 7.5 log CFU/mL for MOI 1 and 1000 (Figure 8a), respectively, after a period of incubation of 6 h. While in the first 4 h of incubation, the inactivation factor was similar for MOI 1 and 1000 (ANOVA, *p* > 0.05), in the first 8 h of incubation, the inactivation factor was higher for MOI 1000 (ANOVA, *p* < 0.05). At a MOI of 1000, after 6, 8, 10, and 12 h of incubation, the reduction in *K. pneumoniae* counts (7.5, 8.1, 7.5, and 6.8 log CFU/mL, respectively) was significantly larger (ANOVA, *p* < 0.05) than that produced with MOI 1 (5.6, 4.8, 4.2, and 4.5 log CFU/mL, respectively). *K. pneumoniae* regrowth was observed after 6 h of incubation. However, at the end of the experiment, the rate of bacterial regrowth with a MOI of 1 and 1000 (reduction of 3.0 and 3.5 log CFU/mL, respectively) was significantly lower (ANOVA, *p* < 0.05) than the one obtained with the bacterial control (Figure 8a). The bacterial reduction for the two MOI values at the end of incubation timeframe (24 h) was statistically similar (ANOVA, *p* > 0.05) (Figure 8a).

Throughout the 24 h of incubation, the bacterial concentration in the control sample (no phage added) increased 5.1 log CFU/mL (Figure 8a), whereas the concentration of phage particles in the phage control sample decreased (0.7 and 1.0 log PFU/mL) for MOI 1 and 1000, respectively (Figure 8b). When phage Kpn31 and its bacterial host were incubated together, one could observe a statistically significant increase in phage concentration (5.9 and 3.0 log PFU/mL, ANOVA, *p* < 0.05) for MOI 1 and MOI 1000, respectively (Figure 8b).

The effect of the MOI value was more pronounced during the first 8–10 h of bacterial inactivation (Figure 8a), with MOI 1000 promoting a higher reduction in bacterial levels relative to MOI 1. However, this difference was significantly reduced by the end of the inactivation assays, with a difference in attained levels of bacteria of only 0.5 log CFU/mL.

### 3.7. Physicochemical Characterization

**Figure 9:**
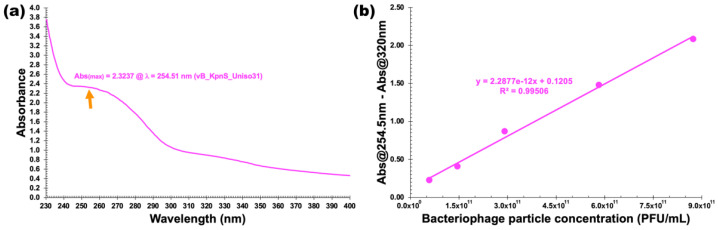
UV–VIS spectral scan of Kpn31 phage particles (**a**) and the relationship between the concentration of phage Kpn31 and its corrected absorbance (**b**).

Physicochemical properties of concentrated suspension of phage Kpn31 (1.165 × 10^13^ virions/mL) were studied via UV–VIS spectral scans and X-ray diffraction (XRD) analysis. Figure 9a shows that the UV–VIS spectral scan of the phage particles suspension produced a maximum absorption at 254.51 nm and a minimum absorption around 245 nm, which is indicative of very low contamination with bacterial cell debris [25,45]. Subtracting the absorbance at 320 nm (where phage chromophores virtually do not absorb light) aimed at correcting for light scattering from phage particles and non-phage particulate contaminants [25,26,45,55]. Fitting the Beer–Lambert equation to the data displayed in Supplementary Table S4, we determined the molar extinction coefficient of the phage particles as ε_Kpn31_ = 2.2877 × 10^−12^ (PFU’s/mL)^−1^ cm^−1^, allowing for calculating the phage particle concentration based on the resulting calibration curve (Figure 9b).

**Figure 10:**
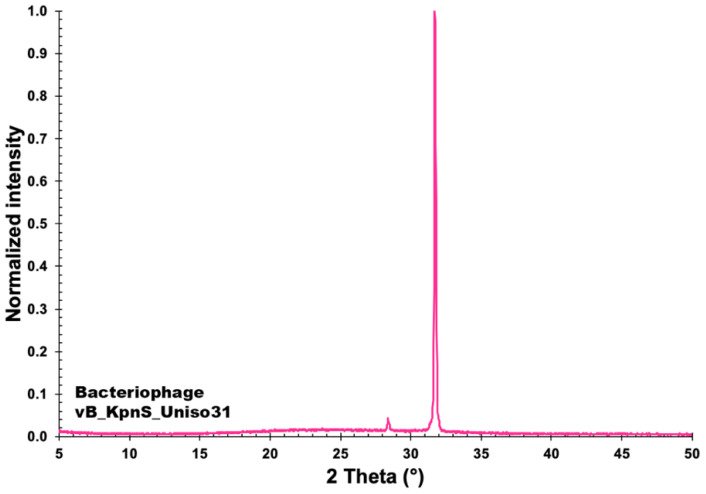
Normalized X-ray diffractogram (XRD) of a sample of the concentrated suspension of phage Kpn31. Diffractogram obtained at an X-ray wavelength of 1.5418 Angstrom.

The normalized X-ray diffractogram of phage Kpn31 is displayed in Figure 10, allowing to observe a generalized amorphous behavior with two peaks of crystallinity. In XRD studies, the Scherrer equation [46] relates the size of sub-micrometre particles, or crystallites, with the widening of a peak in a diffractogram, and it is used for determining the size of the particles of crystals as well as the size and shape of small crystalline regions. The diffractogram of bacteriophage Kpn31 exhibited very small noise, with very well-defined peaks in the region of 28.00 ≤ 2θ ≤ 33.00, with quite a tall peak at 2θ = 31.70°, probably related to external structures of the isolated phage particles. Using the Scherrer equation (see Section 2.14.), the crystallites observed in the X-ray diffractogram (Figure 10) had the probable sizes of τ_2θ = 28.38°_ = 85.65 nm and τ_2θ = 31.70°_ = 43.16 nm. These values are in close agreement with previous results published for *Pseudomonas aeruginosa* phages [25]. The produced sharp peaks in the diffraction pattern of phage Kpn31 by the scattering of X-rays by crystalline structures may thus serve as a signature [56] for the phage particles.

## 4. Discussion

*K. pneumoniae* is among the leading bacterial species that cause opportunistic nosocomial infections and is considered a worldwide threat to public health [57]. There is increasing incidence of multi-drug resistant *K. pneumoniae* strains with the accompanying loss of effectiveness of the antimicrobial therapies [58]. Thus, there is an urgent need for new therapeutic strategies for the control of this pathogen, and the use of bacteriophages has resurged as a useful approach to treat MDR *K. pneumoniae* infections [23].

This work focused on the isolation and characterization of a phage that infects MDR *K. pneumoniae* strains. Phage Kpn31 has a siphovirus-like morphotype according to transmission electron microscopy and was classified as *Demerecviridae* or *Sugarlandvirus* based on its genome sequence. Dozens of *K. pneumoniae* phages, spanning most of the Caudovirales families, have been isolated from various sources, such as sewage, wastewater, river, and marine water [23,59,60]. However, not a lot of *K. pneumoniae* phages currently classified as *Demerecviridae* have been reported [60]. Given that *Demerecviridae* phages present a siphovirus morphotype, we reasoned that this number might increase as genome sequences of *K. pneumoniae* phages previously classified as *Siphoviridae* are available. For instance, the *K. pneumoniae* phage Sugarland was previously classified as *Siphoviridae* [23]. Kpn31 has a genome of 113,444 bp, which is quite similar to the genome size of phages from *Sugarlandvirus* genus. Moreover, the Kpn31 genome does not encode known toxins or antimicrobial resistance genes, which is a required property for phage therapy applications [61]. The taxonomic lineage of phage vB_KpnS_Uniso31 was thus established as: Viruses › Duplodnaviria › Heunggongvirae › Uroviricota › Caudoviricetes › Caudovirales › *Demerecviridae* › *Sugarlandvirus* [62].

The phage Kpn31 genome does not appear to encode depolymerases-related sequences, as has been observed for several *Klebsiella* phages, including *Sugarlandvirus* [60]. Accordingly, Kpn31 lysis plaques does not present the typical halos around the clear zones of lysis observed for depolymerase-producing phages [63].

One (if not the major) advantage of antimicrobial phage treatment lies in the high specificity of phage particles, even though they should be able to promote the lysis of the majority of strains of a given bacterial species [23,64,65,66]. Phage Kpn31 infected about 46% of the MDR *K. pneumoniae* clinical isolates tested (Table 3). However, phage Kpn31 did not infect other bacterial strains of the *Enterobacteriaceae* genera, nor other strains belonging to other bacterial families. Among the 26 strains of MDR *K. pneumoniae* derived from clinical isolates, 11 were susceptible to Kpn31 on spot test, out of which two strains scored low EOP (Table 3), indicating that phage Kpn31 particles were able to bind to these strains and promote bacterial death either via an abortive infection or lysis from without [67,68], but were unable to yield sufficient progeny virions. On the other hand, six MDR *K. pneumoniae* strains exhibited a high EOP (>50%). An EOP of 100% means that every phage particle adsorbing to a susceptible bacterial cell can inject its DNA and produce a lysis plaque under ideal conditions [67]. Zurabov and Zhilenkov (2021) observed that two *K. pneumoniae* phages (vB_KpnS_FZ10 and vB_KpnP_FZ12) were active against a high percentage of *K. pneumoniae* strains (57% and 71%, respectively) [69]. However, the lytic activity of the phages vB_KpnS_FZ41 and vB_KpnM_FZ14 was only about 29% [69]. In another study, the lytic spectra of 32 *K. pneumoniae* phages showed that phages of the *Siphoviridae* and *Podoviridae* families lysed about 7–15% of strains, and only one phage of the *Podoviridae* family was effective against 22% of the strains tested [59]. Phages of the *Myoviridae* family were active against 4–22% of *K. pneumoniae* strains [59]. Phage Kpn31 showed a rather high lytic activity against *K. pneumoniae* strains, which confirms its potential for prophylaxis and for the treatment of bacterial infections caused by this bacterium. In the future, new phages need to be isolated and tested together with phage Kpn31 in order to produce a cocktail with a broader spectrum of activity against *K. pneumoniae* and other human pathogenic bacteria.

Before applying phages to control bacterial pathogens, the dynamics of phage–host replication should be well characterized in vitro. The first step in the phage infection process is the adsorption of the phage virion onto a susceptible bacterial cell [43,70,71,72]. Such adsorption can be usually described by mass-action kinetics [44], implicitly assuming equal influences of the density of bacterial host cells and the adsorption rate [70,71,73]. Hence, a microenvironment with a high bacterial host cell density may mimic a phage displaying a high adsorption rate. The adsorption of a phage particle to its bacterial host cell is a combination of diffusion, biochemical interactions at the host cell surface, and reaction-induced three-dimensional conformational changes in surface receptor proteins [44,74,75]. According to Bull et al. (2014) [76], in phages used to treat infections, 70% or more of the phage particles should be adsorbed onto the target host cells in the first 10 min, and the adsorption constant should be 10^−8^–10^−9^ mL/min. Approximately 90% of phage Kpn31 particles adsorbed to *K. pneumoniae* CCCD—K001 cells after 10 min, and 100% of the phage particles adsorbed after 30 min, which confirms its potential for the treatment and prevention of bacterial infections. The adsorption rate of phage Kpn31 particles (1.700 × 10^−9^ PFU^−1^ CFU^−1^ mL^−1^ h^−1^) closely agrees with the results published by Zurabov et al. (2021) [69] for *K. pneumoniae* phages (phages vB_KpnS_FZ10, vB_KpnS_FZ41, vB_KpnP_FZ12, and vB_KpnM_FZ14).

The growth parameters of Kpn31 phage particles showed a burst size of 9.12 PFU/host cell, allowing to conclude that the phage replicates well in *K. pneumoniae* CCCD-K001 with a short latency period (30 min). The latent period of phage Kpn31 correlates well with published data on *K. pneumoniae* phages [59,69,77]. The latent period of phage Kpn31 was similar to that of phage vB_KpnS_FZ10 (30 min), phage vB_KpnP_FZ12 (30 min), phage vB_KpnM_FZ14 and phage vB_KpnS_FZ41 (35 min) [69], phage Z (24 min) [77], and phage vB_KpnM_KP15 and phage vB_KpnM_KP27 (25 min) [59]. The burst size of phage Kpn31 was similar to that of phage vB_KpnM_KP15 and phage vB_KpnM_KP27 (10–15 PFU/host cell) [59], but lower than that of phages vB_KpnS_FZ10 (80 ± 2 PFU/host cell), vB_KpnP_FZ12 (80 ± 7 PFU/host cell), vB_KpnS_FZ41 (118 ± 3 PFU/host cell), and vB_KpnM_FZ14 (120 ± 5 PFU/host cell) [69]. Despite its relatively low burst size, phage Kpn31 promoted a significant decrease in the growth of *K. pneumoniae*, but its effect only started after 4 h of incubation. *K. pneumoniae* was effectively inactivated by phage Kpn31, reaching a maximum inactivation of 5.6 log CFU/mL and 7.5 log CFU/mL after 6 h of incubation at MOI 1 and 1000, respectively. After this time, although some host cells were not inactivated by phage Kpn31, after phage treatment, the cell growth was much slower. Between 8 h and 24 h of treatment with phage Kpn31 at MOI 1 and 1000, the concentration of host cells was significatively lower than that observed for the control.

At MOI 1, the phage concentration increased by approximately 10-fold (from 10^5^ to 10^6^ PFU/mL, Figure 8b), whereas at MOI 1000, the phage concentration increased by approximately 10,000 fold (from 10^8^ to 10^12^ PFU/mL, Figure 8b) over the first 4 h of treatment. Yet, the bacterial growth was essentially the same for the control and MOI 1 culture, and almost the same for the MOI 1000 culture, from 10^5^ to 10^10^ and from 10^5^ to 10^9+^ CFU/mL, respectively, during the first 4 h of treatment (Figure 8a). To produce a phage titer of ca. 10^12^ PFU/mL (Figure 8b) with a burst size of 9.12 PFU/host cell, it would have required the infection of approximately 10^11^ bacterial cells during that timeframe.

An important issue when inactivating bacteria with phages is the appearance of bacterial cells resistant to the phage, a phenomenon particularly common to *K. pneumoniae* [23,55,64,66,78,79,80]. Phage Kpn31 did not fully prevent the regrowth of bacteria during the treatment. According to several studies, the drawback of bacterial resistance to the phage can be surpassed by using phage cocktails [55,66,79,81,82,83,84,85].

Increasing the MOI from 1 to 1000 for phage Kpn31 significantly increases the treatment efficiency. Although bacterial reduction with phage Kpn31 was higher at a MOI of 1000 (with a decrease of 7.5 log CFU/mL after 6 h of incubation (Figure 8a)), the initial dose of phage Kpn31 was not essential because of the self-perpetuating nature of phage particles, which was revealed by a high increase of phage titers along with bacteria at MOI 1. The number of phage particles during the 12 h of incubation in the presence of the host at a MOI of 1 increased more (by 5.9 log PFU/mL) than at a MOI of 1000 (by 3.0 log PFU/mL) (Figure 8b). In line with the kinetic theory of antibacterial phage treatment, which states that MOI may be of the utmost importance for the effectiveness of bacterial inactivation by the phage, with the inactivation of pathogenic bacterial host increasing side by side with the value of MOI or starting sooner at bigger values of MOI, other researchers [83,85] have reported that well defined initial amounts of phage may not be absolutely necessary due to the self-replicating nature of phages. In fact, this is probably the biggest advantage of antibacterial phage treatment when compared to the conventional antimicrobial therapy. In addition, high MOI values may be even deleterious for the success of antibacterial phage treatment, as the target bacterial host might be killed before replicating the phage particles. This may occur when a bacterium is infected simultaneously by a large number of phage particles and lysis occurs due to the presence of lysins in large concentrations, a phenomenon known as “lysis from without” [67,68]. In fact, by the end of phage treatment, the difference in bacterial inactivation was only of 0.5 log CFU/mL for both MOI values.

The phage Kpn31 host range was initially evaluated by spot test to assess if it was able to produce clear plaques on strains of different bacterial genera and of MDR *K. pneumoniae* clinical isolates. However, the spot test may produce false positives due to bacterial cell lysis, without them being infected by the phage [86], either due to a high number of phage particles adsorbing to the bacterial cell or to residual endolysins present in the bacteriophage suspensions [68,87,88]. In addition, the isolated phages will not always infect only the isolation host, infecting in general bacterial hosts that display the same types of receptors at the surface as the isolation host [89].

The high bacterial inactivation efficiency of phage Kpn31 combined with the safety of the phage and its efficiency against *K. pneumoniae* strains paves the way for new studies, especially in vivo studies, aiming at the development of strategies to control infections caused by *K. pneumoniae.* The results of this study highlight the importance of isolating, characterizing, and testing the effectiveness of new phages to inactivate bacteria in clinically relevant settings.

## Figures and Tables

**Table 1:** Genomic features of the phage Kpn31 genome.

Feature	Phage vB_KpnS_Uniso31 (Phage Kpn31)
NCBI/Genbank accession number	ON637170
Genome size	113,444 bp
Number of PE reads in the final assembly	491,866
Average sequencing coverage	1000×
GC content	45.3%
tRNA genes	14
Protein-coding genes (CDS) predicted	188
With function assigned	44/188 (23.4%)
Hypothetical/unknown function	144/188 (76.6%)
Hypothetical with predicted functional description	54/144 (37.5%)

**Table 2:** Phages that clustered together with phage Kpn31 in the network analysis performed.

NCBI Accession	Description	Genome Size (bp)	% Nucleotide Identity	Query Cover	Reference
			Relative to Knp31 Genome		
KX845404	Klebsiella phage vB_Kpn_IME260	123,490	95.89	95	[49]
MG459987	Klebsiella phage Sugarland	111,103	96.72	85	[50]
MK521907	Klebsiella phage vB_KpnS_FZ41	106,104	95.74	87	[51]
MK630230	Klebsiella phage Spivey	110,659	93.84	84	unpublished
MN101228	Klebsiella phage KPN4	108,916	96.82	89	unpublished
MN163280	Klebsiella phage KpGranit	122,710	96.68	95	[52]
MN434094	Klebsiella phage AmPh_EK80	112,215	95.82	82	[53]
MN434096	Klebsiella phage JIPh_Kp127	113,671	95.35	93	[53]
MN478377	Bacteriophage Eos	109,015	76.89	24	unpublished
MT380195	Klebsiella phage vB_Kpn_B01	113,227	96.14	96	[54]
MW713578	Klebsiella phage Shaphc-TDM-1124-4	112,841	95.93	96	unpublished
MZ079855	Klebsiella phage vB_Kpn_3	112,003	89.56	77	unpublished

**Table 3:** Host range of phage Kpn31 determined on 39 bacterial strains. Numbered strains of *K. pneumoniae* were derived from clinical isolates from human patients (see Supplementary Table S3 for antibiotic susceptibility profile). Clear lysis zone (+) and no-lysis zone (−) on the spot tests. An efficiency of plating (EOP) value of 100% was considered for the host strain *K. pneumoniae* CCCD—K001. n.d.—not determined.

Bacterial Strains	Source	Phage Kpn31	
		Spot Test	EOP (%)
*Escherichia coli* CCCD—E003	Collection	−	n.d.
*Salmonella enterica* CCCD—S004	Collection	−	n.d.
*Pseudomonas aeruginosa* CCCD—P004	Collection	−	n.d.
*Proteus mirabilis* CCCD—P001	Collection	−	n.d.
*Enterococcus faecalis* CCCD—E002	Collection	−	n.d.
*Bacillus subtilis* CCCD—B010	Collection	−	n.d.
*Staphylococcus epidermidis* CCCD—S010	Collection	−	n.d.
*Staphylococcus aureus* CCCD—S009	Collection	−	n.d.
*Acinetobacter baumanii* ATCC 16606	Collection	−	n.d.
*K. pneumoniae* CCCD—K001	Collection (Urine)	+	**100**
*K. pneumoniae* strain 1	Anal swab	+	**84.64**
*K. pneumoniae* strain 2	Urine	+	**71.23**
*K. pneumoniae* strain 3	Urine	−	n.d.
*K. pneumoniae* strain 4	Anal swab	+	**89.21**
*K. pneumoniae* strain 5	Urine	+	**70.18**
*K. pneumoniae* strain 6	Urine	−	n.d.
*K. pneumoniae* strain 7	Urine	−	n.d.
*K. pneumoniae* strain 8	Anal swab	+	**45.36**
*K. pneumoniae* strain 9	Anal swab	−	n.d.
*K. pneumoniae* strain 10	Anal swab	+	**9.54**
*K. pneumoniae* strain 12	Femur secretion	+	**0.90**
*K. pneumoniae* strain 13	Urine	+	**59.64**
*K. pneumoniae* strain 14	Urine	−	n.d.
*K. pneumoniae* strain 15	Hemoculture	−	n.d.
*K. pneumoniae* strain 16	Catheter tip	+	**12.89**
*K. pneumoniae* strain 17	Urine	−	n.d.
*K. pneumoniae* strain 18	Anal swab	−	n.d.
*K. pneumoniae* strain 19	Urine	−	n.d.
*K. pneumoniae* strain 20	Urine	−	n.d.
*K. pneumoniae* strain 21	Anal swab	−	n.d.
*K. pneumoniae* strain 22	Urine	−	n.d.
*K. pneumoniae* strain 23	Anal swab	−	n.d.
*K. pneumoniae* strain 24	Anal swab	+	**55.12**
*K. pneumoniae* strain 26	Urine	−	n.d.
*K. pneumoniae* strain 29	Anal swab	+	**23.66**
*K. pneumoniae* strain 30	Anal swab	−	n.d.

## Data Availability

The phage genome sequence described in this work has been deposited in GenBank NCBI (National Center for Biotechnology Information) under accession number ON637170.

## Supplementary Materials

The following supporting information can be downloaded at: https://www.mdpi.com/article/10.3390/pharmaceutics14071421/s1. Table S1: Antibiotics susceptibility of *Klebsiella pneumoniae* strains derived from clinical isolates from human patients. Table S2: Data utilized to determine the molar extinction coefficient of phage Kpn31. Table S3: Annotation of phage Kpn31 genome. Table S4: Core protein clusters from the 12 phages closest to Kpn31 by the network analysis.
