# Supplementary material for: Isolation and Molecular Characterization of a Novel Lytic Bacteriophage That Inactivates MDR Klebsiella pneumoniae Strains
Source: Pharmaceutics. 2022 Jul 6;14(7):1421. doi: 10.3390/pharmaceutics14071421 (PMC9324672; doi:10.3390/pharmaceutics14071421)
Supplement: Supplementary file 1 [file pharmaceutics-14-01421-s001.zip › pharmaceutics-1781589-supplementary/Table S1_Antibiotics susceptibility of Klebsiella pneumoniae strains.pdf]

Table S1: Antibiotics susceptibility of *Klebsiella pneumoniae* strains derived from clinical isolates from human patients.

Clinical Isolate		Tested antibiotics																						
**Strain**	**Origin**	**NAL**	**AMI**	**AMC**	**AMP**	**CFL**	**CPM**	**CRO**	**CRX**	**CXA**	**CIP**	**ETP**	**GEN**	**MER**	**NIT**	**NOR**	**PPT**	**TRI**	**CAZ**	**COL**	**IPM**	**TGC**	**ASB**	**CFO**
**1**	Anal swab#	R	S	S	R	R	R	R	R	R	R	S	S	S	R	R	S	S	-	-	-	-	-	-
**2**	Urine	R	S	S	R	S	S	S	S	S	R	S	S	S	R	R	S	S	-	-	-	-	-	-
**3**	Urine	R	S	R	R	R	R	R	R	R	R	R	R	R	R	R	R	R	-	-	-	-	-	-
**4**	Anal swab	-	S	-	R	-	R	R	R	R	R	R	R	R	-	-	R	-	R	R	R	R	R	R
**5**	Urine	S	S	S	R	S	S	S	S	S	S	S	S	S	*	S	S	S	-	-	-	-	-	-
**6**	Urine	R	S	I	R	R	R	R	R	R	S	S	S	S	*	S	S	R	-	-	-	-	-	-
**7**	Urine	S	S	S	R	S	S	S	S	S	S	S	S	S	R	S	S	S	-	-	-	-	-	-
**8**	Anal swab	-	S	-	R	-	R	R	R	R	R	S	R	S	-	-	S	-	R	S	S	*	R	S
**9**	Anal swab	-	S	-	R	-	R	R	R	R	R	R	R	R	-	-	R	-	R	R	R	R	R	R
**10**	Anal swab	-	S	-	R	-	R	R	R	R	R	R	R	R	-	-	R	-	R	S	R	R	R	R
**12**	Femur secretion	-	S	-	R	-	R	R	R	R	R	S	R	S	-	-	S	-	R	S	S	*	R	S
**13**	Urine	R	S	R	R	R	R	R	R	R	R	R	S	R	R	R	R	R	-	-	-	-	-	-
**14**	Urine	R	S	S	R	R	R	R	R	R	R	S	S	S	*	R	S	R	-	-	-	-	-	-
**15**	Hemoculture	-	S	-	R	-	R	R	R	R	R	R	R	R	-	-	R	-	R	S	R	R	R	R
**16**	Catheter tip	-	S	-	R	-	R	R	R	R	R	R	R	R	-	-	R	-	R	R	R	*	R	R
**17**	Urine	R	S	S	R	S	S	S	S	S	S	S	S	S	*	S	S	R	-	-	-	-	-	-
**18**	Anal swab	-	S	-	S	-	S	S	*	*	S	S	S	S	-	-	S	-	S	S	S	R	*	*
**19**	Urine	R	S	R	R	R	R	R	R	R	R	R	R	R	R	R	R	R	-	-	-	-	-	-
**20**	Urine	R	S	R	R	R	R	R	R	R	R	S	S	S	R	R	R	R	-	-	-	-	-	-
**21**	Anal swab	-	S	-	R	-	R	R	R	R	R	R	R	R	-	-	R	-	R	S	R	R	R	R
**22**	Urine	R	S	R	R	R	R	R	R	R	R	R	S	R	R	R	R	R	-	-	-	-	-	-
**23**	Anal swab	-	*	-	R	-	R	R	R	R	R	R	R	R	-	-	R	-	R	S	R	R	R	R
**24**	Anal swab	-	S	-	R	-	R	R	R	R	R	S	R	S	-	-	*	-	R	S	S	*	R	S
**26**	Urine	R	S	R	R	R	R	R	R	R	R	S	S	S	S	R	*	R	-	-	-	-	-	-
**29**	Anal swab	-	S	-	R	-	R	R	R	R	R	R	R	R	-	-	R	-	R	S	R	*	R	R
**30**	Anal swab	-	S	-	R	-	R	R	R	R	R	S	S	S	-	-	*	-	R	S	S	*	R	S

Abbreviations of tested antibiotics: **NAL:** nalidixic acid; **AMI:** amikacin; **AMC:** amoxicillin / clavulanic acid; **AMP:** ampicillin; **CFL:** cephalothin; **CPM:** cefepime; **CRO:** ceftriaxone; **CRX**: cefuroxime; **CXA**: cefuroxime acetyl; **CIP:** ciprofloxacin; **ETP:** ertapenem; **GEN:** gentamicin; **MER**: meropenem; **NIT:** nitrofurantoin; **NOR:** norfloxacin; **PPT:** piperacillin / tazobactam; **TRI:** trimethoprim/sulfamethoxazole; **CAZ:** ceftazidime; **COL:** colistin; **IPM:** imipenem; **TGC:** tigecycline; A**SB:** ampicillin sulbactam; **CFO:** cefoxitin. **S:** sensitive; **R:** resistant; ***:** intermediate; **-:** not tested. #extended-spectrum beta-lactamases (ESBL) producing-strain
