# Supplementary material for: Isolation and Molecular Characterization of a Novel Lytic Bacteriophage That Inactivates MDR Klebsiella pneumoniae Strains
Source: Pharmaceutics. 2022 Jul 6;14(7):1421. doi: 10.3390/pharmaceutics14071421 (PMC9324672; doi:10.3390/pharmaceutics14071421)
Supplement: Supplementary file 1 [file pharmaceutics-14-01421-s001.zip › pharmaceutics-1781589-supplementary/Table S2_Data for the molar extinction coefficient.pdf]

Table S2: Data utilized to determine the molar extinction coefficient of phage Kpn31.

$V_{concentrated\ phage\ suspension}$ (μL)	$V_{dilution}$ (μL)	PFU	PFU/mL	$Abs_{254.5\ nm}$	$Abs_{320\ nm}$	$Abs_{254.5nm}$-Abs_{320nm}
10	2000	$1.1645x10^{11}$	$5.8225x10^{10}$	0.3512	0.1252	0.2260
25	2000	$2.9113x10^{11}$	$1.45563x10^{11}$	0.6667	0.2589	0.4078
50	2000	$5.8225x10^{11}$	$2.91125x10^{11}$	1.3113	0.4425	0.8688
100	2000	$1.1645x10^{12}$	$5.82250x10^{11}$	2.3767	0.8966	1.4801
150	2000	$1.7468x10^{12}$	$8.73375x10^{11}$	3.5000	1.4177	2.0823
